# Review of Biomimetic Engineering in the Electrolyte for Aqueous Batteries

**DOI:** 10.3390/ma18184356

**Published:** 2025-09-18

**Authors:** Haoshen Xu, Haoqi Yang, Dawei Sha, Xu Dong

**Affiliations:** Institute of Technology for Carbon Neutralization, College of Electrical, Energy and Power Engineering, Yangzhou University, Yangzhou 225127, China; haoshenxu0824@gmail.com (H.X.); yanghq@yzu.edu.cn (H.Y.); shadw@yzu.edu.cn (D.S.)

**Keywords:** biomimetic electrolyte, aqueous batteries, mimetic, solid electrolyte interphase, antifreeze-protein, ion-channel-mimic

## Abstract

Aqueous batteries, which replace flammable organic electrolytes with water, offer advantages such as intrinsic safety, low cost, and environmental friendliness, making them well-suited to the energy storage needs driven by the increasing proliferation of renewable energy. However, their widespread adoption is hampered by the narrow electrochemical stability window of water, dendrite growth on metal anodes, and various parasitic interfacial reactions. This review proposes a unified three-part framework for biomimetic electrolytes—SEI-mimetic, antifreeze-protein-mimetic, and ion-channel-mimetic—corresponding to three mechanistic strands—water activity regulation, interfacial mechanics, and sub-nanometer transport—to organize and compare various strategies. This paper systematically reviews and evaluates the latest advances in biomimetic electrolytes. It discusses these three biomimetic concepts and their applications in different battery chemistries (monovalent and multivalent metal systems, as well as aqueous redox-flow batteries). It also proposes a roadmap and engineering thresholds for both basic research and commercialization.

## 1. Introduction

Intermittent renewable energy sources have seen significant development to meet the growing global energy demand in an environmentally sustainable manner. To ensure a stable energy supply, large-scale energy storage systems (LSESSs) are essential for storing these variable energy resources. Over the past few decades, organic-ion batteries—such as lithium-ion and sodium-ion batteries—have played a critical role in portable electronics and electric vehicles due to their high energy and power densities. However, their use of flammable and toxic organic electrolytes poses safety and environmental concerns, making them less suitable for large-scale energy storage applications [1].

Against this backdrop, aqueous batteries (ABs) have received intense interest because water-based electrolytes have the merits of non-flammability, high ionic conductivity (~10^−2^ S cm^−1^), and low materials cost [2,3], including aqueous monovalent-ion batteries (Li^+^, Na^+^), aqueous multivalent-ion batteries (Zn^2+^, Mg^2+^, Al^3+^), and aqueous redox-flow batteries. However, the large-scale deployment of ABs is still hampered by three fundamental bottlenecks:

(i) Narrow electrochemical window. The thermodynamic H_2_/O_2_ split limits the aqueous stability window to 1.23 V; practical cell voltages are therefore capped in the range of 1–2 V, severely curbing gravimetric and volumetric energy density [4].
(ii) Metal-anode instability. Metallic Zn and Mg suffer from hydrogen evolution, corrosion, and non-uniform ion flux, culminating in dendritic growth that jeopardizes cycling safety and lifetime [5].
(iii) Conventional aqueous electrolytes suffer from poor thermal stability. Below 0 °C, they tend to freeze, severely restricting ion transport. At temperatures above 60 °C, side reactions become more pronounced, and water loss due to evaporation accelerates, compromising electrolyte integrity and battery performance [6,7].

To address these challenges, various strategies have been explored. “Water-in-salt” and deep eutectic electrolytes have extended the electrochemical stability window to 2–3 V; however, their high viscosity and cost remain significant barriers to large-scale application [4]. To overcome these limitations, researchers have turned to biomimetic electrolyte engineering. By mimicking natural systems—such as biological membranes, ion channels, and ice-binding proteins—it is possible to design interphases that regulate ion flux, broaden the effective stability window, and maintain ionic conductivity under extreme temperatures.

Recent exemplars include a self-assembled lithium perfluorobutanesulfonate (FBS^−^) bilayer that lowers the Zn nucleation overpotential from 42 mV to 21 mV and delivers stable Zn‖Cu plating/stripping for 880 h with an average Coulombic efficiency of 99.9% at 5 mA cm^−2^ with an areal capacity of 5 mAh cm^−2^ [8]. When the same additive is applied in Zn‖Zn_0.25_V_2_O_5_·H_2_O full cells, ~91.57% of the initial capacity is retained after 1200 cycles at a current density of 0.5 A g^−1^ [8]. A nacre-like mesoporous polydopamine sheath, by contrast, enables 1500 dendrite-free Zn cycles (5 mA cm^−2^, 1 mAh cm^−2^) [9], while oxidized g-C_3_N_4_ quantum-dot antifreeze mimics maintain Zn‖Zn integrity for 1000 h at −30 °C and preserve 91.48% capacity in Zn‖NVO cells after 5000 cycles −30 °C [10].

**Figure 1:**
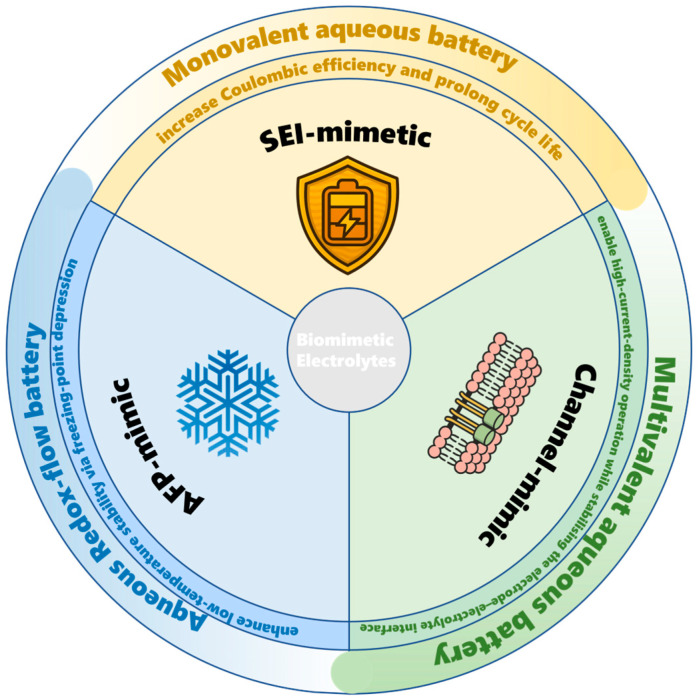
Biomimetic electrolyte taxonomy highlighting SEI-mimetic, antifreeze-protein (AFP)-mimic, and channel-mimic strategies.

Why is a new synthesis needed now? Since 2020, terms such as “SEI-mimetic”, “AFP (antifreeze-protein)-mimic” and “channel-mimic” have proliferated, but their metrics and mechanistic rationales remain fragmented. A unified framework is therefore required to (i) benchmark disparate strategies, (ii) extract transferable design rules and (iii) highlight cost or scale barriers. Figure 1 summarizes these three bio-inspired routes, and the following sections will examine each in turn.

Unlike previous reviews that primarily listed materials or reviewed single systems, this review proposes and adopts a unified tripartite framework for biomimetic electrolytes SEI-mimetic, AFP-mimic, and ion-channel-mimetic, organizing the field into three mechanistic strands (regulation of water activity, interfacial mechanics, and sub-nanometer transport), enabling comparison of quantitative thresholds and transferable design criteria for each strategy.

SEI-mimetic. Lamellar or bilayer artificial interphases can enable solid-like Li^+^/Zn^2+^ conduction (σ ≈ 10^−8^–10^−5^ S cm^−1^) while delivering an out-of-plane shear modulus G > 6 GPa—corresponding to a Young’s modulus E > 9 GPa assuming a Poisson’s ratio ν ≈ 0.3—satisfying the Monroe–Newman criterion for dendrite suppression [11,12]. Polydopamine nanomembranes have already demonstrated E ≈ 13 GPa [13], while natural nacre represents a high-performance benchmark with E ≈ 60–70 GPa [14]. According to the Monroe–Newman instability criterion, the shear modulus of the SEI layer must be at least several times that of the metal anode to suppress the crack of SEI induced by the volume change upon metal-ion plating and stripping. Furthermore, for thin layers of 10–50 nm, if the ion conductivity of σ ≥ 10^−7^–10^−6^ S cm^−1^ and iR drop ≤ 5–25 mV under j ≈ 5 mA cm^−2^, the potential will not be pushed into the side reaction zone, allowing both rapid ion transport to homogenize the ion concentration and electrical field, thereby achieving uniform nucleation and deposition [15].

AFP-mimic. Ice-binding additives or polymer networks depress crystallization to ≤−30 °C, yet retain ionic conductivity ≥ 10^−4^ S cm^−1^ and a cation transference number t^+^ ≥ 0.4 (e.g., oxidized g-C_3_N_4_ quantum dots: t^+^ ≈ 0.46). For the AFP-mimic, diffusion and solvation dynamics slow significantly at subzero temperatures. If σ (ionic conductivity) is too low or a cation transference number t^+^ is too small, the combined effects of concentration polarization and ohmic polarization can rapidly increase the polarization voltage and induce side reactions. For an electrolyte thickness of L ≈ 100 µm, a κ of ≈10^−4^ S cm^−1^ corresponds to a ΔV of ≈0.1 V at a current density of j ≈ 1 mA cm^−2^, which remains within a manageable range. A t^+^ ≥ 0.4 reduces ion accumulation and local pH fluctuations, maintaining reversible low-temperature cycling [10].

Channel-mimic. Aligned nanochannels with effective diameters d < 1 nm—such as the 6.7–11.6 Å pores in COO^−^-functionalized covalent organic frameworks (COFs)—enable size- and charge-selective ion transport. Molecular dynamics simulations reveal that water confined within sub-nanometer slits exhibits a significantly disrupted hydrogen-bonding network, which reduces the energy barrier for cation desolvation [16]. This mechanism is likely at play in the COO^−^-COF separator reported by Yan et al. [17]. At this scale, the first hydration layer is partially stripped off; and the fixed negative charge of the pore wall (such as COO^−^) further inhibits the crossing of anions and water molecules through Donnan repulsion, thereby achieving “size + charge” dual selection and explaining why the COF 6.7–11.6 Å pore can take into account both selectivity and flux.

In this review, Section 2 explores biomimetic electrolytes tailored for monovalent-ion systems (Li^+^, Na^+^). Section 3 extends the discussion to multivalent chemistries (Zn^2+^, Mg^2+^, Ca^2+^), while Section 4 focuses on advances in aqueous redox-flow batteries. Each section concludes with concise key takeaways, highlighting breakthroughs, open questions, and cost considerations. The review concludes with a techno-economic perspective and an integrated research roadmap to guide future development and commercialization efforts.

## 2. Biomimetic Electrolytes for Aqueous Monovalent-Ion Batteries

Aqueous monovalent-ion batteries (AMIBs) utilize alkali–metal ions (Li^+^, Na^+^) as charge carriers in water-based electrolytes. Due to their single positive charge, these ions possess weaker hydration shells and higher diffusion coefficients compared to divalent ions such as Zn^2+^ and Mg^2+^. As a result, they exhibit lower desolvation energy barriers and faster bulk ion transport [2,3,18]. Within monovalent cations, the solvation strength follows Li^+^ > Na^+^ > K^+^ because the effective Lewis acidity/charge density decreases from Li^+^ to K^+^. Consequently, hydrated radii and desolvation barriers are largest for Li^+^ and smallest for K^+^, while diffusion coefficients generally trend K^+^ > Na^+^ > Li^+^ in aqueous media [18]. These differences motivate ion-channel designs and weakly solvating matrices to be tailored to cation-specific hydration energetics. Leveraging abundant and low-cost Na^+^ and K^+^ salts, AMIBs can be constructed using high-capacity insertion-type anodes—such as hard carbon or NASICON-type NaTi_2_(PO_4_)_3_—paired with Prussian blue analogs (PBAs, E ≈ +0.9 V vs. SHE) or polyanionic phosphates (E ≈ +0.6–0.75 V vs. SHE) as cathodes. These combinations yield full-cell voltages in the range of ~1.5–1.8 V [19,20,21]. This design circumvents the use of metallic Li, Na, or K anodes, which react violently with water and are thus restricted to short-lived “water-in-salt” electrolyte demonstrations [22].

Despite recent progress, three major challenges continue to hinder the commercialization of AMIBs: (i) hydrogen evolution and electrode corrosion at the negative electrode; (ii) persistent side reactions that generate by-products and gradually increase interfacial impedance [23,24]; and (iii) poor low-temperature performance, particularly near 0 °C, where electrolyte freezing elevates viscosity and obstructs ion transport—not due to inherently slow cation kinetics [6]. While concentrated “water-in-salt” electrolytes can alleviate issues (i) and (ii), they often suffer from extremely high viscosities—exceeding 100 mPa·s when ionic–liquid co-salts are introduced [7]. These limitations highlight the urgent need for biomimetic electrolyte strategies that can simultaneously ensure safety, maintain fast ion transport, and enable low-temperature operation.

**Figure 2:**
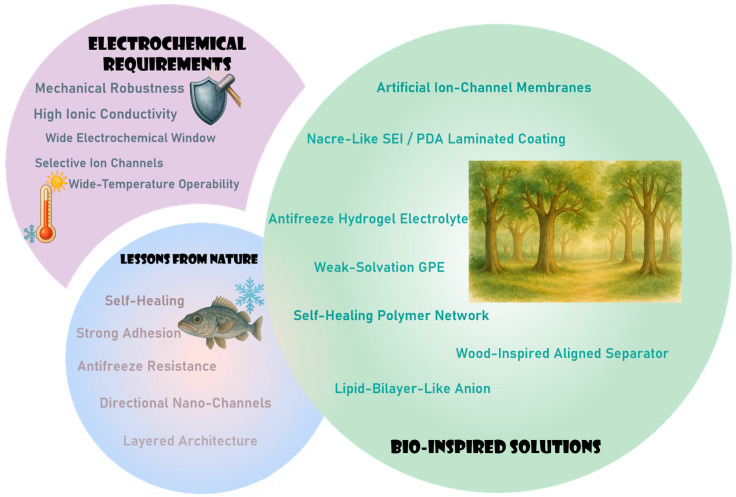
Intersecting design axes linking electrochemical requirements, lessons from nature, and bio-inspired solutions [5,8,9,10,13,14,25,26,27,28,29,30,31,32,33].

Aqueous monovalent-ion batteries (AMIBs) offer high specific capacities, but their practical implementation is hindered by three critical limitations: (i) surface corrosion and parasitic hydrogen evolution, which reduce Coulombic efficiency; (ii) a narrow electrochemical stability window, leading to limited energy density; and (iii) poor ion mobility at low temperatures due to water crystallization. To overcome these challenges, recent research has drawn inspiration from biological systems—such as membranes, antifreeze proteins, and ion channels—to engineer the following: (a) ultra-thin lamellar interphases that homogenize ion flux; (b) weakly solvating or cryoprotective gels that preserve electrolyte fluidity at temperatures as low as −40 °C; and (c) sub-nanometer ion channels that selectively conduct cations while excluding bulk solvent. The following subsections explore how these biomimetic concepts have been applied to Li^+^, Na^+^ systems, followed by a synthesis of cross-cutting insights (Figure 2).

### 2.1. Shared Bottlenecks and Design Logic

Aqueous monovalent-ion batteries (AMIBs) offer the dual advantages of resource abundance and high ionic conductivity, typically exceeding 10^−2^ S cm^−1^ at room temperature [2]. However, parasitic hydrogen and oxygen evolution reactions limit the practical cell voltage to approximately 1.5–2.0 V [4]. Additionally, water freezes at 0 °C, while highly concentrated “water-in-salt” electrolytes suffer from elevated viscosities (η > 100 mPa·s) at low temperatures [7]. To address these interrelated limitations, biomimetic strategies are being developed across three hierarchical levels:

Membrane-like interphases—Artificial solid–electrolyte interphases (SEIs) inspired by lipid bilayers that regulate and homogenize ion flux;

Antifreeze or weak-solvation matrices—Polymer networks that disrupt hydrogen bonding, reducing water activity and enhancing low-temperature performance;

Channel-mimetic nanostructures—Aligned sub-nanometer pores (d < 1 nm) that enable size- and charge-selective ion transport [16].

### 2.2. Lithium-Based Aqueous Batteries

Aqueous lithium-ion batteries (ALIBs) typically employ intercalation-type anodes such as Li_4_Ti_5_O_12_ (LTO) or NASICON-type LiTi_2_(PO_4_)_3_ (LTP), paired with spinel cathodes like LiMn_2_O_4_ (LMO) [25]. However, their practical deployment is constrained by three interrelated challenges: (i) a narrow electrochemical stability window (~1.9–2.0 V vs. Li^+^/Li), which limits energy density; (ii) Mn dissolution and proton-induced degradation at the cathode; and (iii) increased viscosity or electrolyte freezing at temperatures below 0 °C. Recent advances in biomimetic electrolyte engineering offer a promising integrated approach to simultaneously expand the stability window, stabilize electrode–electrolyte interfaces, and maintain ionic conductivity under low-temperature conditions.

**Figure 3:**
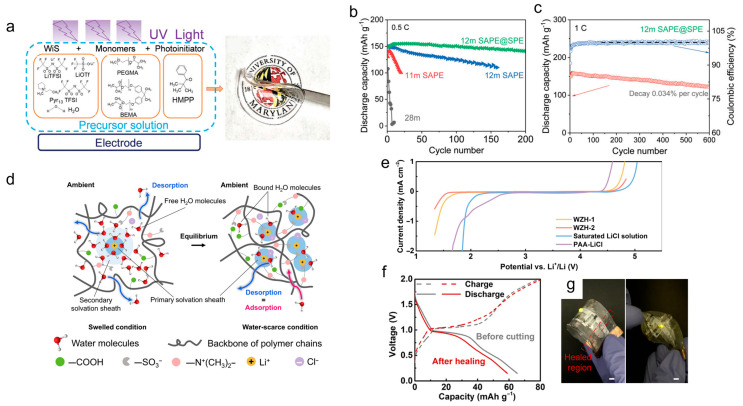
(**a**) Chemical structure of the compositions used to synthesize solid-state aqueous polymer electrolyte networks, and a prototype of a typical SAPE network; (**b**) cycling stability of the LMO‖LTO full cell in 28 m WiSE, 11 m SAPE, 12 m SAPE, and 12 m SAPE@SPE electrolytes at 0.5 °C; (**c**) cycling stability and Coulombic efficiency of the LMO‖LTO full cell in 12 m SAPE@SPE electrolyte at 1 °C [25]. Reproduced from [25] with permission from the Royal Society of Chemistry. (**d**) Illustration of the lithium solvation shell in the zwitterionic hydrogel under swelled and water-scarce conditions; (**e**) comparison of ESWs of WZHs with different MEDSAH/AA monomer ratios; (**f**) GCPL profiles for the pristine battery (gray color) and after a prototype is cut and put at 70 °C for 10 min for the healing process (red color); (**g**) optical image showing the operation of electronic system under mechanical stresses such as folding (left) and twisting (right) after cutting and the subsequent self-healing process [26]. Reproduced from He et al. [26], distributed under the Creative Commons Attribution-NonCommercial 4.0 license (CC BY-NC 4.0, https://creativecommons.org/licenses/by-nc/4.0/, accessed on 24 August 2025).

Cytoplasm-inspired “water-in-salt” polymer electrolytes (WiSPEs) have been created. Zhang et al. [25] developed a UV-curable poly (acrylate) network integrated into a highly concentrated 12 m LiTFSI solution (~65 wt%) (Figure 3a). The polymer chains act as macromolecular crowding agents, mimicking the dense environment of cytoplasm, thereby disrupting the hydrogen-bond network and lowering water activity. This approach extends the electrochemical stability window to approximately 3.8 V, enabling LMO‖LTO full cells to deliver an energy density of 151 Wh kg^−1^ (at 0.5 °C, based on active materials) with excellent cycling stability, maintaining 99.97% Coulombic efficiency over 600 cycles (Figure 3b,c). Desert-plant-inspired “water-scarce” hydrogels have also been created. He et al. [26] designed a fluorine-free hydrogel electrolyte with only 19 wt% water, inspired by succulents that retain moisture within hierarchical pore structures (Figure 3d). This biomimetic confinement strategy extends the anodic stability limit to 3.11 V vs. Li^+^/Li (Figure 3e). Soft V_2_O_5_‖LMO pouch cells assembled with this hydrogel demonstrated ~90% capacity retention after mechanical deformation (bending and cutting) and 500 cycles at ambient temperature, highlighting the synergistic benefits of water confinement and a self-healing polymer matrix (Figure 3f,g).

Biomimetic electrolyte design for ALIBs converges on three principles: (1) selective hydration control via macromolecular crowding to widen the voltage window; (2) hierarchical confinement in water-scarce gels to preserve ionic conductivity below 0 °C while suppressing parasitic reactions; and (3) dynamic polymer networks—offering self-healing in hydrogels and high toughness in WiSPEs—to provide mechanical resilience for flexible devices. Future efforts should (i) quantify the lifecycle cost of bio-sourced polymers, (ii) couple operando neutron imaging with multiscale simulation to map Li^+^ flux, and (iii) benchmark > 2 mAh cm^−2^ areal loadings under freeze–thaw cycling to accelerate translation to grid-scale and wearable markets.

### 2.3. Sodium/Potassium-Ion Aqueous Batteries

Sodium/Potassium ions have abundant reserves in the earth’s crust, and considering the difficulty of obtaining them, sodium/potassium ions can be obtained directly from seawater or soda ash, which significantly reduces the cost of obtaining raw materials. It has common problems with aqueous lithium-ion batteries, such as a narrow electrochemical stability window, large water radius, interface instability, slow desolvation, etc. These problems have become the main factors restricting the development of aqueous sodium/potassium ions.

In this context, the team of Yan et al. was inspired by the Na/K biological channel and proposed a bio-inspired design of a Na-ion conduction channel in the covalent organic framework of a quasi-solid-state sodium battery, which increased the originally narrow electrochemical stability window to 5.32 V. It was demonstrated that the battery has fast reaction kinetics at 60 mA g^−1^ and 25 ± 1 °C, low polarization voltage and stable cycling performance at 1000 cycles with a capacity of 0.0048% per cycle, and a final discharge capacity of 83.5 mAh g^−1^ [17].

**Figure 4:**
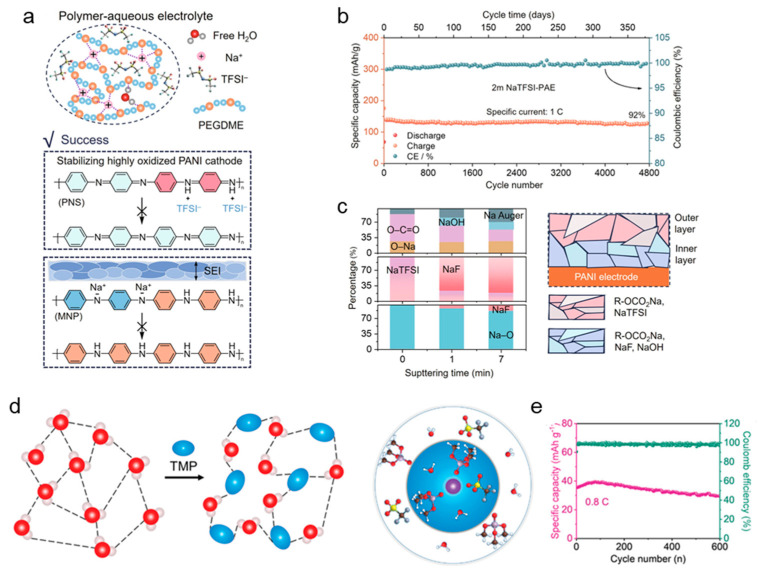
(**a**) Stable all-polymer ASIBs enabled by 2 m NaTFSI polymer-aqueous electrolyte; (**b**) cycling performance and Coulombic efficiency in 4800 cycles (381 days); (**c**) ratio of different Na salts of SEI after different sputtering times (0, 1, and 7 mins), and SEI structure on PANI anodes [27]. Adapted from Ref. [27] under the Creative Commons Attribution 4.0 license (CC BY 4.0, https://creativecommons.org/licenses/by/4.0/, accessed on 24 August 2025). (**d**) Illustration of the evolution of the K^+^ primary solvation sheath in K-H_2_O-TMP_4.9_ electrolyte, and schematic illustration of the interaction between water and TMP; (**e**) cycling performance and Coulombic efficiency of the full cell at 0.8 °C [34]. Adapted from Ref. [34] with permission from Wiley-VCH GmbH. © 2023 Wiley-VCH GmbH. All rights reserved.

The research of Hong et al.’s team targeted the problem of interface instability. The research aimed to reduce water activity and construct a stable SEI to extend the cycle life. They reported a polymer aqueous electrolyte that stabilizes the redox products of the polymer electrode by regulating the dissolution layer and forming a solid electrolyte mediator. In their article, they mentioned a specific electrolyte formulation: 2 m NaTFSI dissolved in a polymer aqueous electrolyte of PEGDME (molecular weight ≈ 450) and H_2_O; in practical applications, 2 m NaTFSI-PEGDME(450)-H_2_O (PAE) was used as the working electrolyte. The PEGDME content was evaluated in the range of 50% to 94 wt% to adjust the H_2_O activity. This system significantly reduces free water activity. On the other hand, a multi-component SEI (Figure 4a,c) with alkyl carbonate (R–OCO_2_Na) as the main component and only a few nanometers of NaF as the inner layer is formed in situ on the electrode surface, thereby effectively isolating the side reaction and maintaining a low impedance interface. The all-polymer hydrated sodium-ion battery they made used polyaniline as a symmetrical electrode. It has a capacity of 139 mAh g^−1^ and an energy density of 153 Wh kg^−1^. After 4800 cycles, it still maintains a capacity of >92% and a Coulombic efficiency of >99.5% (Figure 4b). It has extremely good performance and lays the foundation for the future field of flexible electronics [27]. Diluted aqueous hybrid electrolytes with regulated core–shell solvation structures retain ~85% capacity after 600 cycles at 0.8 °C and still deliver stable performance up to 4 °C in K-ion cells. Specifically, they employed an aqueous-aprotic mixed electrolyte of 1.6 mol L^−1^ potassium trifluoromethanesulfonate in a water/trimethyl phosphate (TMP) solvent, denoted as K-H_2_O-TMP_4.9_ (Figure 4d,e) [34].

A comprehensive analysis of the aforementioned reports shows that the abundant sodium reserves and low mining costs provide aqueous sodium-ion batteries with natural economic and resource advantages. However, their large hydration radius, narrow electrochemical window, and interfacial instability remain core bottlenecks restricting performance improvement. The Yan and Hong teams have proposed two different strategies to address these challenges, focusing on “increasing voltage” and “extending lifespan”, respectively. These strategies outline a complementary and synergistic technical roadmap for developing aqueous sodium-ion batteries with high energy density and long cycle life.

### 2.4. Comparative Summary

Across Li and Na aqueous batteries, three converging principles emerge: (i) lamellar or bilayer SEIs redistribute ion flux and mechanical stress [12], (ii) weak-solvation or cry-protective gels retain conductivity below 0 °C [10], and (iii) sub-nanometer channels balance desolvation energy with selectivity [16,17]. For fair cross-study comparison, the lifespans quoted below follow the unified benchmark protocol in Table 1. Yet, gaps persist: most demonstrations remain < 50 mAh, the cost of COF membranes is high [17], and unified testing protocols for freeze–thaw cycling are lacking [6]. Addressing these pain points will determine whether biomimetic monovalent cells can graduate from coin cells to grid-level modules.

Under the unified baseline introduced in this section, SEI-mimetic/weak-solvation gels (e.g., WiSPE and water-scarce hydrogels) trade wider aqueous ESW and low-temperature fluidity against salt content and viscosity; they excel in voltage window and mechanical compliance in ALIB full cells [25,26]. Channel-mimetic COFs provide size/charge selectivity and low polarization in Na systems but face membrane synthesis/scale-up cost constraints [17]. Polymer-aqueous electrolytes (PAE) that form R–OCO_2_Na/NaF interphases push cycle life to 4800 with CE > 99.5% in all-polymer Na cells, highlighting a clear longevity advantage [27]. In practice, one prioritizes voltage window (WiSPE), low-T/flexibility (water-scarce gels), or selectivity and de-solvation (COF channels) depending on device targets. Table 1 summarizes the normalized lifetime and test conditions.

## 3. Biomimetic Electrolytes for Multivalent Aqueous Batteries

Unlike monovalent systems that shuttle a single charge carrier (Li^+^, Na^+^), multivalent aqueous batteries (MVABs) utilize ions such as Zn^2+^ and Mg^2+^, which transfer two or more electrons per redox event. This higher charge density offers a theoretical advantage in volumetric capacity and cost-effectiveness, as zinc and magnesium are earth-abundant and commercially available at just 1–3 $ kg^−1^ [35]. In addition, the use of aqueous electrolytes eliminates flammable solvents, enhancing safety for grid-scale and wearable applications. However, the strong interactions between multivalent cations and water molecules lead to sluggish desolvation kinetics, while aggressive interfacial reactions—such as hydrogen evolution and corrosion—compromise long-term cycling stability. Biomimetic strategies, inspired by systems such as nacre shells, ice-binding proteins, and biological ion pumps, have recently emerged as promising approaches to address these challenges, as discussed in the sections below.

### 3.1. Context, Challenges and Bio-Inspired Rationale

Multivalent aqueous batteries—primarily based on Zn^2+^ and Mg^2+^ chemistries—offer the advantage of transferring two electrons per redox event with minimal fire risk. However, they face two fundamental molecular-scale challenges: (i) strong solvation, where highly charged cations carry bulky hydration shells (n_H_2_O ≈ 6–9) and polarize surrounding water molecules, increasing the desolvation barrier and limiting ion transport kinetics; and (ii) interfacial instability, as this polarization also catalyzes hydrogen evolution and pitting corrosion, leading to dendrite formation or passivating by-products well before commercial lifetimes are achieved [5,35,36]. As reiterated in Figure 2, the key design requirements—mechanical robustness, selective ion transport, and wide-temperature operability—remain critical for multivalent systems but must now address the challenges imposed by higher charge density.

In recent years, researchers have actively used biomimetics to address these intertwined problems. For example, structures imitating shell layers have been well applied in distributing mechanical stress and can exclude free water. Inspiration from wooden cell walls and sea urchin spines has led to the creation of low-tortuosity channels, which are effective in balancing ion flux. Antifreeze protein strategies will weaken the hydrogen bond network and reduce the freezing point to ≤−30 °C.

### 3.2. Zinc: The Crucible for Biomimetic Design

Elemental zinc combines a moderate anode potential (~−0.76 V vs. SHE) with earth-abundant availability and rapid two-electron redox kinetics. However, it is susceptible to parasitic hydrogen evolution in mildly acidic or neutral electrolytes due to its relatively low overpotential for the hydrogen evolution reaction (HER), and its passive ZnO/Zn(OH)_2_ film is unstable in chloride- or sulfate-rich environments [37]. Comparative degradation studies reveal that magnesium and its alloys, despite having a more negative standard potential, often produce larger volumes of hydrogen gas under physiological pH conditions because of aggressive anodic dissolution. In contrast, zinc exhibits an intermediate HER rate among multivalent metals [38,39].

**Figure 5:**
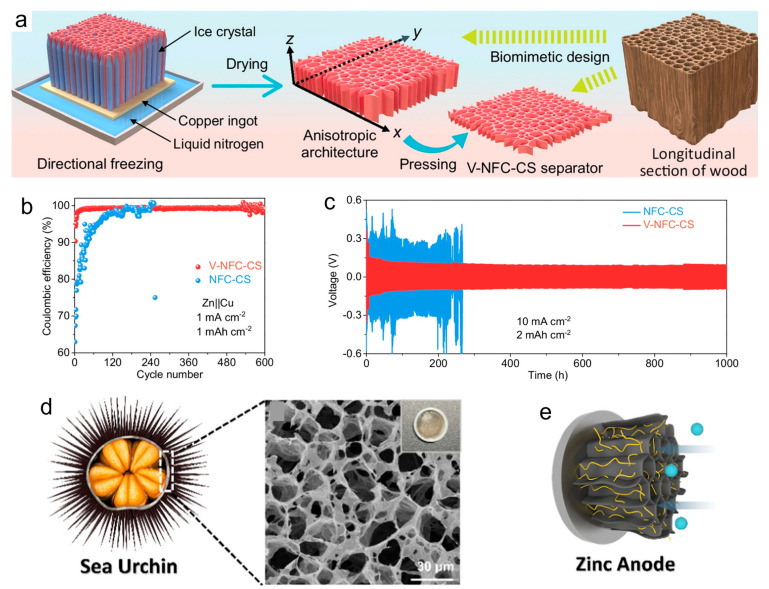
(**a**) Schematic illustration of the preparation procedure of the V-NFC-CS separator; (**b**) CE vs. cycle number of Zn‖Cu cells with two different separators at 1 mA cm^−2^ and 1 mAh cm^−2^; (**c**) GCD profiles of Zn‖Zn cells with two different separators at 10 mA cm^−2^ and 2 mAh cm^−2^ [28]. Reproduced from Ma et al. [28], under the Creative Commons Attribution 4.0 license (CC BY 4.0, https://creativecommons.org/licenses/by/4.0/). (**d**) Cross-sectional anatomy of a sea urchin, with its shell structure in the rectangle; (**e**) illustration diagram of the aligned GO-SA-coated zinc anode, with facile zinc ions infiltration [29]. Adapted with permission from Chen et al. [29]. Copyright (2025) American Chemical Society.

**Figure 6:**
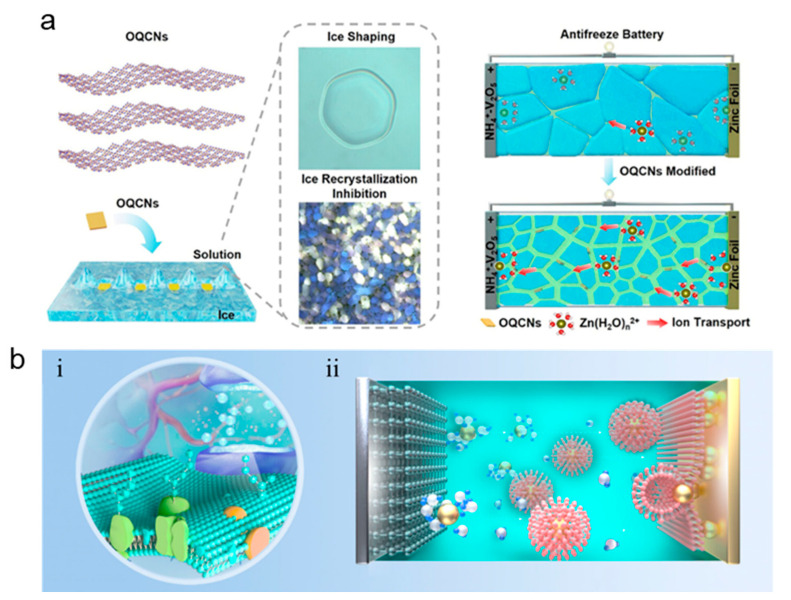
(**a**) Mechanism of bioinspired synthetic AFPs mimics of OQCNs for ice growth inhibition and schematic diagram of low-temperature operation of antifreeze aqueous zinc-ion battery and functions of OQCNs [10]. Adapted with permission from Zhu et al. [10]. Copyright (2025) Wiley-VCH GmbH. (**b**) Schematic diagram of the lipid bilayer and the mechanism of a biomimetic electrolyte and characterization of the biomimetic electrolyte: (**i**) lipid bilayer structure in biology and (**ii**) mechanism of a biomimetic electrolyte in the battery [8]. Adapted with permission from Wu et al. [8]. Copyright (2025) American Chemical Society.

**Figure 7:**
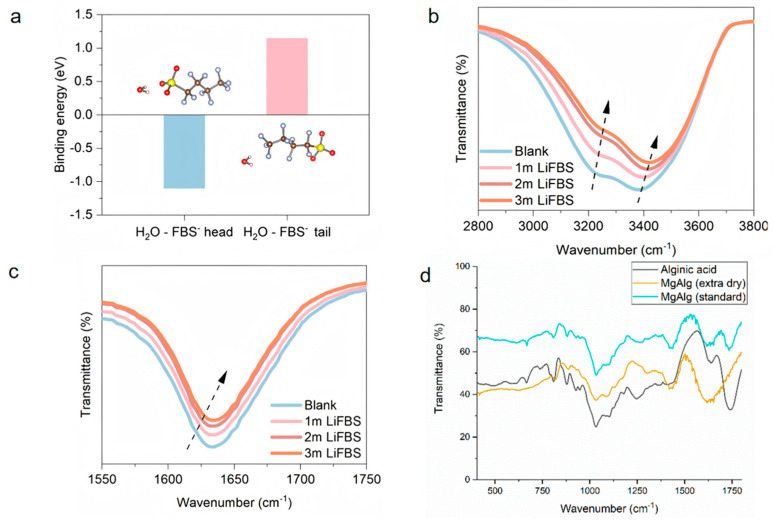
(**a**) Binding energy between the H_2_O–FBS head and H_2_O–FBS tail. (**b**,**c**) FTIR analysis of the water activity limited by ionic clusters formed by FBS and H_2_O. Arrows indicate the direction of peak shift [8]. Adapted with permission from Wu et al. [8]. Copyright (2025) American Chemical Society. (**d**) FTIR spectra of the Mg–Alg samples against the Alg–acid [42]. Reproduced from Kwakernaak et al. [42], under the Creative Commons Attribution 4.0 license (CC BY 4.0, https://creativecommons.org/licenses/by/4.0/).

In response to the corrosion and dendrite phenomenon faced by aqueous zinc ions, many teams have paid attention to this pain point and have made different degrees of improvement through bionics. Ai et al.’s team used mesoporous polyketamine (2D-mPDA) platelets as building blocks to construct a reliable superstructure solid electrolyte interphase for a stable Zn anode. They deposited a 2D mesoporous PDA film; catechol coordination accelerated the desolvation of Zn^2+^, achieving 1500 dendrite-free cycles at 5 mA cm^−2^ and a Coulombic efficiency of 99.8% (5 mA cm^−2^, 1 mAh cm^−2^, 25 °C) [9]. Ma et al. conducted relevant research on wood channel membranes. They used ice casting to prepare a 23 µm thick nanocellulose chitosan membrane. Its ordered nanotubes can buffer the local pH and stabilize Zn‖Zn for 1000 h at 10 mA cm^−2^ (8 mA cm^−2^, 4 mAh cm^−2^, 25 °C) (Figure 5a–c) [28]. The service life of the Zn‖Zn battery is extended while improving the cyclability of the full battery. Inspired by sea urchin spines, the team of Chen et al. laminated a graphene oxide/alginate layer with a pore size of <1 nm; the introduction of this biomimetic layer of ion-conductive natural polymer greatly alleviated the aqueous side reaction, and the nucleation overpotential was reduced from 42 mV to 21 mV. The soft-pack battery with a capacity of 7 Ah still retained 92.7% of its capacity after 500 cycles (10 mA cm^−2^, 1 mAh cm^−2^, 25 °C) (Figure 5d,e) [29]. Tan et al. stabilized the zinc anode by adding 2-pyridinecarboxylic acid (PCA) to the electrolyte; PCA provides multi-site Zn^2+^ coordination. A symmetric Zn‖Zn battery using PCA/ZnSO_4_ electrolyte can stably cycle for 3500 h at 1 mA cm^−2^, 1 mAh cm^−2^, and 298 K, and can still cycle for 2000 h at 2 mA cm^−2^, 2 mAh cm^−2^, and 298 K, showing excellent long-term cycling performance [40]. The team of Zhu et al. was inspired by the antifreeze protein (AFP) mechanism and oxidized g-C_3_N_4_ quantum dots to simulate antifreeze protein. By controlling the ice crystal morphology and inhibiting the growth kinetics, it effectively performed biomimetic functions and improved its antifreeze performance. It maintained the integrity of Zn‖Zn for 1000 h at −30 °C and supported > 5000 low-temperature cycles (3 mA cm^−2^, 1 mAh cm^−2^, −30 °C) (Figure 6a) [10]. Wu et al.’s team [8] was inspired by the lipid bilayer structure in biology and showed excellent reversibility. Lithium perfluorobutanesulfonate (FBS^−^) can self-assemble into a lipid bilayer on the Zn surface, excluding free H_2_O but allowing Zn^2+^ to pass through. Figure 6b shows the transition from micelle to bilayer and its hydrophobic interface, which reduces the Zn nucleation overpotential from about 42 mV to about 21 mV and achieves 880 h of dendrite-free cycling at 5 mA cm^−2^ with an average Coulombic efficiency of 99.91%. As shown in Figure 7a–c, the strong coordination selectivity between FBS^−^ and water (binding energy calculation) and the FTIR changes in the O–H/C=O vibration region indicate that the FBS-induced ion clusters significantly weaken the hydrogen bond network of water, thereby effectively reducing water activity and providing precursor chemicals for SEI formation. Ye et al.’s team was inspired by the biological role of bamboo parenchymal cells (BPC); a biomimetic electrolyte additive was introduced to enhance the performance of RAZIBs. In this study, the porous bamboo fiber additive smoothed the interfacial pH, extending the battery life to 3000 h. The half-cell showed a high average Coulombic efficiency (99.67%) in 380 cycles at 5 mA cm^−2^, demonstrating the scalability of biological resources [41].

Across lamellar SEIs, directional nanochannels, and ice-binding additives, the unifying objective is to regulate hydrated-ion desolvation while minimizing local pH fluctuations. Polydopamine (PDA) and FBS^−^ approaches act directly at the metal–electrolyte interface; wood- or urchin-inspired architectures guide ion flux within the separator; and AFP mimics enhance ion transport kinetics at sub-zero temperatures. Remaining challenges include operando mapping of Zn^2+^ solvation dynamics at areal loadings exceeding 5 mAh cm^−2^, as well as comprehensive life-cycle cost analyses of bio-derived polymers relative to DOE cost targets expressed in USD kWh^−1^.

### 3.3. Magnesium: Emerging Beneficiaries

Magnesium ions offer the advantages of abundant resources and low cost. Compared to lithium, they are approximately 1000 times more abundant and can be produced at low cost through the electrolysis of seawater brine. Because each Mg^2+^ can transfer two electrons, its theoretical volumetric capacity is high, thus possessing significant energy potential. Similarly to lithium and zinc, the commercialization of rechargeable magnesium batteries depends primarily on the electrolyte’s compatibility with magnesium metal. In aqueous systems, this paradox manifests itself: the extremely strong hydration energy (ΔG_hyd_ ≈ −1500 kJ mol^−1^) creates a high desolvation barrier for Mg^2+^, while polarized water molecules accelerate hydrogen evolution and corrosion. Therefore, constructing biomimetic chelating networks or interfacial coatings that can both weaken the hydration shell and inhibit hydrogen evolution is a key breakthrough for aqueous magnesium batteries.

Markus C. Kwakernaak and others were inspired by algae and extracted polysaccharide alginate from algae to use it as an aqueous polymer electrolyte in magnesium-ion batteries, thereby effectively improving the conductivity between electrodes and constructing a renewable, non-toxic, and biodegradable hydrogel network. In Figure 7d, FTIR of the Mg-Alg gel shows the formation and differentiation of Mg^2+^–carboxylate coordination, revealing the coupling law of the solvation/coordination-interface in multivalent systems. In this system, Mg^2+^ is weakly coordinated with the polymer chain, which not only retains mobility but also significantly reduces the free water activity. The mechanism is similar to the concept of “water-in-salt”. In the test of ionic conductivity using EIS, the 2 wt% magnesium electrolyte showed a conductivity of 1.8 × 10^−3^ S cm^−1^, which is an excellent performance [42].

In the aqueous magnesium-ion system, its room-temperature conductivity of 1.8 mS/cm and self-generated conductive film verified the feasibility of the biomimetic strategy in aqueous magnesium-ion batteries and provided a green electrolyte design route for the research of aqueous magnesium-ion batteries.

### 3.4. Mechanistic Synopsis

Taken together, biomimetic multivalent electrolytes unify structure, chemistry, and function: lamellar or β-sheet frameworks provide mechanical backpressure; catechol and alginate ligands modulate solvation structures; and antifreeze protein (AFP)-inspired additives tailor the hydrogen-bond network. Recent advances have achieved Coulombic efficiencies exceeding 99% (Zn) and demonstrated cryogenic operation down to −30 °C.

If we want to move biomimetic multivalent aqueous batteries from the laboratory to megawatt-hour energy storage applications, the next steps should focus on four aspects. (1) Precisely control pore size, surface charge, and tortuosity to match the hydration dynamics of Zn^2+^, Mg^2+^, and Al^3+^. (2) Quantify the cost of bio-sourced materials such as silk, alginate, and cellulose ($ kg^−1^) and compare it with the DOE energy cost target ($ Wh^−1^). (3) In situ mapping: integrate neutron imaging with molecular dynamics and phase field modeling to visualize hydrated ion transport under realistic areal loading conditions. (4) Standardize protocols: under conditions with areal capacities greater than 5 mAh cm^−2^ and undergoing more than 100 freeze–thaw cycles, uniformly report Coulombic efficiency, voltage efficiency, and temperature changes to ensure fair and comparable benchmarking. Once we form a systematic engineering solution in these links, biomimetic multivalent aqueous batteries can take another step forward from the laboratory to large-scale deployment to truly serve the grid.

Benchmark protocol. Unless otherwise noted, all symmetric Zn‖Zn and full-cell data quoted below are recalculated using a reference areal capacity of *Q*_areal = 1 mAh cm^−2^, at room temperature (25 °C) and a nominal current density of *J* = 5 mA cm^−2^. Lifetimes reported in hours are converted to equivalent cycle numbers using N = It/(2*Q*_areal). Full metadata—including current density, areal loading, temperature, electrolyte composition, and cell geometry—are compiled in Table 2.

From Table 2, it is not difficult to see that the layered/bilayer SEI (such as 2D-mPDA) directly reshapes the ion flux and interface mechanics. It has the advantages of low overpotential and interphase robustness, but its coating process is complex [9]. The directional nanochannels in the wood separator can buffer the local pH value. It is outstanding in low tortuosity and anisotropic conductivity, but it faces a thickness-impedance trade-off [28]. The sea urchin-shaped ion sieve can significantly reduce the nucleation overpotential (42→21 mV). Its advantages are device-level scalability and the alleviation of interfacial side reactions, but it still faces the limitations brought by the cost of multi-layer lamination and the increased interface complexity [29]. Ligand additives (such as PCA) can achieve uniform ion flux and inhibit hydrogen evolution through multi-site coordination. Their advantages are low cost, “add and use”, and long life. However, they are limited by the possibility of ligand consumption during long-term operation, requiring long-term monitoring of concentration and compatibility [40]. The AFP simulant maintained Zn‖Zn for 1000 h at −30 °C, and the full battery was cycled at low temperature for >5000 cycles. It is operable under subzero conditions and maintains low-temperature ion transport limitations, but its cross-system compatibility still needs to be verified [10]. Mg-alginate hydrogel, as a green and biodegradable material, has a green, safe, and renewable matrix, but it is still in the early stages of performance and needs further verification on high-load devices. Compared with (local) high-concentration electrolyte/local eutectic (LHCE/LEE), LHCE often relies on inert diluents to maintain a high coordination environment locally in non-aqueous systems; however, it is more challenging to achieve LHCE in an environment where diluent selection and compatibility are limited in aqueous systems. In contrast, Mg-alginate achieves a localized high coordination effect with less diluent through “ion clusters/coordination networks” at the molecular level, which is an alternative path for material–structure synergy [42].

## 4. Biomimetic Electrolytes in Aqueous Redox-Flow Batteries

An aqueous redox-flow battery (RFB) stores energy in two liquid electrolytes—anolyte and catholyte—that circulate through an external electrochemical cell stack. Power output scales with the cell stack area, while energy capacity scales independently with the volume of electrolyte stored in external tanks, enabling flexible system designs ranging from tens of kilowatts to multi-megawatts [43,44]. Because all active species remain dissolved, RFBs avoid the volume-change fatigue common in solid electrodes and can achieve calendar lifetimes exceeding 15 years with virtually unlimited cycle life [44,45]. The use of aqueous electrolytes eliminates flammable solvents, enhancing intrinsic safety, while the ability to top up or rebalance electrolytes simplifies maintenance and end-of-life recycling [46,47]. Although current commercial systems are dominated by all-vanadium chemistries, nature-derived organic molecules are emerging as cost-effective, tunable alternatives—paving the way for biomimetic electrolyte engineering.

### 4.1. Context and Design Rationale

Large-scale stationary storage—ranging from sub-hour grid balancing and multi-hour renewable buffering to backup power for data centers—requires systems in which power and energy can be scaled independently [43]. Aqueous organic redox-flow batteries (AORFBs) are well suited to this architecture, yet their practical deployment faces three interrelated challenges: (i) crossover of active species through ion-exchange membranes; (ii) molecular instability due to side reactions such as nucleophilic attack and radical dimerization; and (iii) low intrinsic conductivity of many water-soluble organics, necessitating high concentrations of supporting salts.

Biomimetic electrolyte design addresses these bottlenecks by drawing inspiration from biological systems, which have evolved efficient strategies for solubility enhancement, self-repair, and selective transport. Nature-derived redox motifs offer chemically stable electron reservoirs; protein-mimetic channels and hydrogels provide size- and charge-selective pathways along with dynamic self-healing; and hydrogen-bond networks regulate proton transfer. Thus, Figure 2 also functions as a conceptual framework for AORFB electrolyte design: the left-hand circle (electrochemical demands) maps onto goals of membrane conductivity and redox stability, while the lower circle (self-healing, antifreeze, and nano-channels) informs the design of polymer matrices and ion-sieving separators discussed below.

### 4.2. Bio-Derived Redox Couples: From Vitamins to Lignin

**Figure 8:**
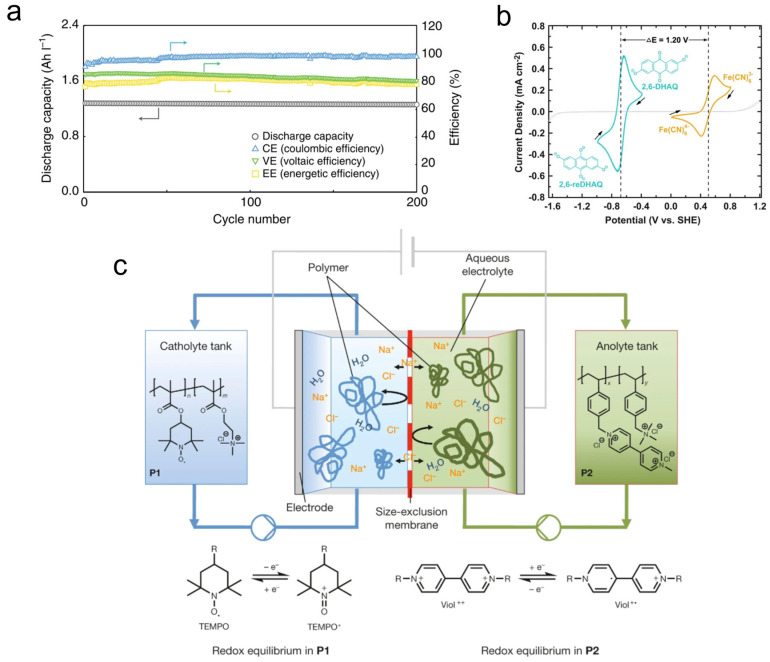
(**a**) Charge–discharge profiles at 1st, 100th, and 200th cycles at 10 mA cm^−2^ [49]. Reproduced from Orita et al. [49], under the Creative Commons Attribution 4.0 license (CC BY 4.0, https://creativecommons.org/licenses/by/4.0/). (**b**) Cyclic voltammogram of 2 mM 2,6-DHAQ (dark cyan curve) and ferrocyanide (gold curve) scanned at 100 mV/s on glassy carbon electrode; arrows indicate scan direction. The dotted line represents the CV of 1 M KOH background scanned at 100 mV s^−1^ on graphite foil electrode [51]. Reprinted with permission from Lin et al. [51]. Copyright (2015) The American Association for the Advancement of Science (AAAS). (**c**) Schematic representation of a polymer-based RFB consisting of an electrochemical cell (which determines the power density) and two electrolyte reservoirs (which determine the storage capacity) [48]. Reprinted with permission from Janoschka et al. [48]. Copyright (2015) Springer Nature.

Aqueous liquid flow battery systems have long been limited by narrow potential windows and plagued by problems such as cycle life. However, biomolecules from vitamins to lignin are naturally compatible under aqueous conditions, and their toxicity and raw material costs are lower than traditional vanadium salts in liquid flow battery systems. Researchers have greatly improved the cycle life and energy density of aqueous organic liquid flow batteries through biomimetic strategies such as flavin mononucleotides, glycosylated anthraquinones, amino acid-modified benzoquinones, and lignin oligoquinones, while the overall cost is lower than that of the vanadium system. Notably, a landmark polymer-based aqueous RFB employing non-corrosive, low-cost electrolytes retained about 80% of its capacity after 10,000 consecutive cycles at 20 mA cm^−2^ in a static cell, highlighting the feasibility of durable water-based organic chemistries (Figure 8c) [48].

To address the high-cost issue, the team of Akihiro Orita et al. reported the use of a flow battery with an aqueous electrolyte based on the sodium salt of flavanone mononucleotide. They used the “two electrons and one proton” isoalloxazine ring of FMN to achieve 200 cycles at 10 mA cm^−2^ (Figure 8a), showing stable cycling performance in a strong base redox-flow battery [49]. In order to reduce the cost of active materials and improve the solubility and cycle life in alkaline systems, Peng et al.’s team [50], based on the natural “glycosidation detoxification” mechanism of plants for anthraquinone toxins, carried out O-glycosidation of 2,6-dihydroxyanthraquinone to synthesize hydrophilic DHAQ Glc on the basis of the 2,6 DHAQ/ferrocyanide alkaline flow battery prototype proposed by Lin’s team (Figure 8b) [51]. Subsequently, Peng et al.’s team introduced tetramethylammonium into the same system to regulate the solvation structure of DHAQ, increasing the available electrolyte concentration to 0.4 M and reducing the capacity decay rate under 0.1 M conditions from 5.34% d^−1^ to 0.65% d^−1^, significantly extending the long-cycle stability of the battery [50]. To overcome the lifespan issue of flow batteries, Pang et al.’s team grafted cysteine onto phenylazine molecules, introducing zwitterion hydration and free radical quenching effects, and maintained 90% of the capacity after 1000 cycles at 300 mA cm^−2^ [52]. Lignin is one of the natural biopolymers on Earth. The team of Monalisa Chakraborty et al. oxidized and cracked kraft paper lignin in KOH to produce a benzoquinone-rich oligomer negative electrode electrolyte; under 50 g L^−1^ (≈0.31 M) and 150 g L^−1^ (≈0.94 M) conditions, it can be stably cycled for >200 cycles when paired with a ferrocyanide positive electrode, with an average Coulombic efficiency of approximately 91% (Figure 7c), demonstrating the potential of lignin-derived quinones as low-cost, sustainable next-generation energy storage systems [53].

These examples reflect a shared principle: preserve the bio-inspired scaffold that imparts water solubility or radical stability while fine-tuning the redox potential through minimal substitution. Trade-offs arise between molecular weight (affecting energy density) and chemical robustness, motivating the membrane innovations discussed below.

### 4.3. Biomimetic Ion-Sieving Membranes

Aqueous flow batteries (RFBs) rely on ion exchange membranes to separate the positive and negative electrolytes. While conventional Nafion membranes offer excellent conductivity, they are very expensive and lack selectivity for organic active molecules, leading to cross-permeation of active species, capacity fading, and energy efficiency loss. Consequently, research is shifting to lower-cost, more selective protein-channel biomimetic membranes. These membranes aim to achieve a “molecular sieving” effect through precise pore size and surface chemistry, significantly reducing transmembrane permeation while maintaining a constant resistance.

The team of Ye et al. reported the preparation of size-selective ion exchange membranes by sulfurization of helical protein fluorine-based microporous polymers and demonstrated their efficient ion screening function in liquid flow batteries. The sPIM SBF membrane reduced the transmembrane flux of K_4_Fe(CN)_6_ and 2,6 DPPAQ by >95% with the sub-nanopores of ≈0.6 nm (0.2–0.7 nm) in the skeleton. At 100 mAcm^−2^, the energy efficiency was about 78% (about 12 percentage points higher than the 66% of Nafion115), and it could be stably cycled for 2100 cycles with a capacity decay of 7.9 × 10^−5^%/cycle. The membrane surface resistance was only 0.82 Ω cm^2^ (Nafion115 was 1.57 Ω cm^2^), confirming that the “protein channel” type precise confinement can significantly improve the efficiency and life of aqueous liquid flow batteries while reducing ionic impedance [30].

To address the pain points of high membrane resistance and poor chemical stability under strong alkaline conditions, the team of Hu et al. prepared a size-selective ion exchange membrane that has excellent ion screening function in liquid flow batteries, significantly improving cycle life and energy efficiency. Cts Cu M (chitosan Cu^2+^ cross-linked composite membrane) relies on nano-confined hydrogen bond channels to achieve a surface resistance of 0.17 Ω cm^2^ at a current density of 320 mAcm^−2^; the alkaline Zn flow battery can still operate continuously for 200 cycles, and the energy efficiency remains at about 80%. This study provides a new and reliable method for alkaline liquid flow batteries at high power [31].

The biomimetic protein channel membrane is another innovation in the field of ion exchange membranes for aqueous flow batteries, providing an excellent solution to the bottlenecks of cost, efficiency, and life. The results of Ye and Hu’s team show that precise pore size control and dynamic coordination can solve the current pain points and provide a new solution for the next generation of high-power, long-life, and low-cost energy storage systems.

### 4.4. Protein-like Supramolecular Matrices

Currently, ion exchange membranes account for a large proportion of the cost of flow battery stacks, are very expensive, and can cause problems such as mold penetration of active materials in various parts and mechanical fatigue. These factors together limit the service life and efficiency of flow batteries. The use of biomimetic mechanisms, such as protein-like supramolecular matrices, can reduce costs while taking into account other functions, such as self-healing or strong mechanical strength and low permeability. The following introduces the research progress in this field.

The team of Hou et al. conducted relevant research on the crack and propagation problems caused by water expansion and pressure accumulation in the cell stack of anion exchange membranes (AEMs). They used a Diels–Alder cross-linked chitosan/benzyl furfuryl network, which could spontaneously re-bond within 10 min at 80 °C, restoring the tensile strength after complete fracture by >90%. In a neutral pH viologen‖TEMPO flow battery, the membrane maintained a Coulombic efficiency of ≥97% and an energy efficiency of approximately 80% for 100 consecutive cycles at 100 mA cm^−2^ [32].

Xi et al.’s team studied ion exchange membranes for vanadium redox-flow batteries (VRFBs). A catechol-rich PDA nanolayer was grown on the surface of sulfonated PEEK to form a dynamic Fe^3+^ catechol bridge, which can suppress the cross-permeation of V^4+^/^5+^ by ≥90%, maintain a Coulombic efficiency of 95–100% at 100 mAcm^−2^, and effectively prevent membrane shedding during 100 charge and discharge cycles in flexible pouch cells. This PDA coating has high mechanical strength and thermal stability [33]. Bionic protein-like supramolecular matrices achieve multiple functions that are difficult to achieve with traditional membranes through the “dynamic reversible bond + sub-nanometer confinement” strategy. The proposed research method not only significantly reduces the risk of transmembrane permeation and mechanical failure but also has lower costs, providing a new answer to breakthroughs in the safety, lifespan, and economics of aqueous flow batteries [33]. Finally, we summarize the test conditions and key performance of each representative work, as shown in Table 3.

### 4.5. Perspective

**Figure 9:**
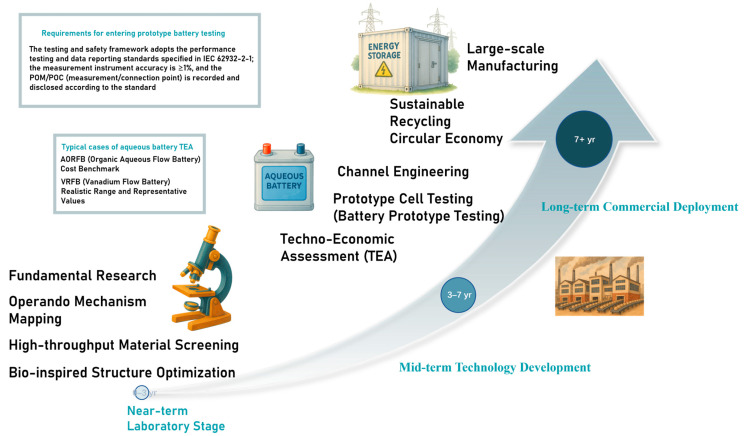
Research and commercialization roadmap for biomimetic aqueous batteries: from laboratory discovery to large-scale deployment [54,55,57].

Figure 9 sketches a time-scale roadmap from laboratory discovery to commercial deployment. There are numerous TEA cases, but here are two classic examples: a cost benchmark for Aqueous Organic Flow Batteries (AORFBs) employs Monte Carlo uncertainty analysis, yielding average capital costs of approximately 674 and 398 €/kWh for 4 and 8 h, respectively. Two LCOS calibers are reported: one accounting for irreversible losses alone and one including the total cost of charging energy (e.g., 4 h, 530~663 €/MWh). The probability range for AORFBs being lower than VRFB capital costs under current conditions is quantified (approximately 16.9–29.6%) [54]. A realistic range and representative values for VRFBs (All-Vanadium Flow Batteries) are based on a device-level TEA framework based on large-scale multi-cell stack parameters and real market prices, directly outputting economic indicators [55]. Bio-derived molecular design has progressively lifted active-material concentration in AORFBs from <0.1 M to the present 1–2 M range, while state-of-the-art systems now demonstrate the following: (i) 99% capacity retention over 100 cycles for flavin mononucleotide cells at 100 mA cm^−2^ [49]; and (ii) 99.98% capacity retention after 500 cycles at 100 mA cm^−2^ in a biomimetic high-capacity phenazine anolyte [56]. Polymerized lignin negolytes further exhibit <0.01% fade per cycle [48]. Despite these advances, three research vectors remain pivotal:

The primary task is to establish precise channel engineering: correlating pore size distribution with the Stokes radius of redox molecules to achieve ion selectivity of ≥95% at an area resistance of <0.3 Ω cm^2^. Lifecycle and cost analyses should also be conducted simultaneously. Current prices for silk protein and chitosan grades are approximately $15–30 kg^−1^; converting this to $ Wh^−1^ and benchmarking it against the DOE’s proposed $75 k Wh^−1^ target will clarify economic feasibility. Furthermore, the large-scale supply of green raw materials is a key step. Scaled production of lignin-derived quinones or chitin-chitosan requires the development of sustainable extraction routes and verification of the biodegradability of the membrane materials.

Concerted progress along these axes could elevate biomimetic AORFBs from pilot stacks to hundred-megawatt installations, closing the loop between renewable generation and resilient, eco-friendly storage.

## 5. Conclusions and Future Outlook

Biomimetic electrolyte engineering—defined as the rational transplantation of biological structures (e.g., lamellae, ion channels) and functional chemistries (e.g., catechol-metal coordination, zwitterionic solvation)—has pushed aqueous batteries well beyond the historical 1.23 V stability limit. Across single and multivalent systems, this strategy has enabled the following: (i) dendrite-free metal deposition with Coulombic efficiencies ≥ 99% [17]; (ii) electrochemical windows approaching ~3 V in water-in-salt electrolytes [4]; and (iii) robust cycling at −30 °C by incorporating antifreeze-protein mimics and nanoconfined water networks [10]. In aqueous organic redox-flow batteries, bio-derived redox species span a broad performance spectrum: flavin mononucleotide systems retain ~99% capacity over 100 cycles [49].

### Future Research Priorities

1. Operando mechanism mapping—By combining liquid cell TEM with soft X-ray microscopy, the migration of hydrated ions, SEI formation, and local stress evolution are simultaneously captured. Molecular dynamics and machine learning potential energy surfaces are then combined to atomically reduce the transient processes of solvation shell rearrangement and hydrogen bond breakage. Phase field and finite element models are then extended to the device scale, ultimately constructing a multi-scale closed loop that reveals the 3D coupling law of “water activity-interface chemistry-mechanical failure”.

2. High-throughput bio-material discovery—Relying on chemical informatics and graph neural networks, functional group vector matching is performed on natural polymer databases such as chitosan, silk fibroin, and lignin, quickly identifying candidate molecules containing fragments such as COO^−^, catechol, or glycoside OH. A microfluidic spectroscopy platform is then used to complete automated testing of solubility, viscosity, and ESW, achieving monthly screening iterations and real-time evaluation of biodegradation and toxicological characteristics to ensure that the material meets green design principles as soon as it is launched.

3. Scalability and techno-economics—Upscale gram-scale syntheses to kilogram batches; build ≥100 Ah stacks that cycle stably at ≥5 mA cm^−2^; and benchmark $ kg^−1^/$ Wh^−1^ cost metrics against the U.S. DOE Long-Duration Storage Shot goal of ≤$0.05 kWh^−1^ levelized cost of storage by 2030 [58,59].

4. Sustainability and safety—Conduct carbon footprint, leaching toxicity, and end-of-life disposal assessments on biopolymer electrolytes; utilize blockchain traceability technology to record raw material sources, production carbon emissions, and recycling flows; and finally, systematically verify thermal runaway and gas escape to provide hardcore safety data for global market access and insurance ratings.

Beyond this, cross-disciplinary evidence highlights electrolyte motifs and manufacturability routes that could accelerate transformation while clarifying remaining bottlenecks. The seawater battery literature has mapped corrosion-resistant chemical and ion-sieving separators for chloride-rich media. These design rules for such separators (graded porosity, antifouling coatings, and slowed Cl_2_ evolution) can be transferred to biomimetic water systems aimed at achieving long-life anodes and selective membranes. Scaling anisotropic channel membranes via roll-to-roll processing while quantifying chloride/halide side reactions within grid-relevant salinity windows is a pressing task [60]. In situ polymerization of poly (ionic liquid) electrolytes offers a practical manufacturing route to increase interfacial density and modulate viscosity/ion transport. Applying this process to zwitterionic, bio-sourced PIL gel formulations has yielded electrolytes that are water-compatible, have low toxicity, and exhibit enhanced mechanical integrity. However, monomer cost, potential cytotoxicity, and recyclability remain limiting factors, motivating efforts to pursue kilogram-scale synthesis accompanied by lifecycle and leachate testing [61]. In summary, the most promising near-term routes are the following: (i) ion-channel-mimicking, halide-tolerant membranes; (ii) in situ polymerized PIL-based gels with zwitterionic/biosourced monomers; and (iii) hierarchically porous, strongly anchored redox interfaces. Immediate obstacles are scale (roll-to-roll membranes, kilogram-scale green monomers), cost (€/kg and €/kWh targets), toxicity (monomer/binder leachates), and long-term stability (salt crossover, catalyst dissolution). Addressing these issues requires standardized prototype gates (areal loading/current density), coupled TEA-LCA, and well-defined durability metrics penalizing halide corrosion and leaching.

5. Closing remark—The convergence of electrochemistry, material science, and biomolecular engineering is forging a pathway towards aqueous batteries that are simultaneously safe, high-performance and environmentally responsible. Interdisciplinary collaboration will be essential for transforming today’s laboratory prototypes into tomorrow’s megawatt-hour-scale assets on a renewables-dominated grid.

## Figures and Tables

**Table 1:** Unified benchmarking of recent biomimetic Li/Na strategies.

Strategy	Metal	*J* mA cm^−2^	*Q* Areal mAh cm^−2^	*T* °C	Life Cycles	Cum. Charge Ah cm^−2^
WiSPE [25]	Li	0.5	0.5	25	600	0.3
Water-scarce hydrogel [26]	Li	—	—	25	500	—
COF channel [17]	Na	—	—	25	1000	—
PAE [27]	Na	—	—	25	4800	—

**Table 2:** Unified benchmarking of recent biomimetic Zn/Mg strategies.

Strategy	Metal	*J* mA cm^−2^	*Q* Areal mAh cm^−2^	*T* °C	Life Cycles	Cum. Charge Ah cm^−2^
2D-mPDA SEI [9]	Zn	5	1	25	1500	1.5
Wood-channel sep. [28]	Zn	8	4	25	1000	4.0
AFP QDs [10]	Zn	1	1	−30	1000	1.0
FBS bilayer [8]	Zn	5	5	25	1230	6.1
Bamboo cellulose [41]	Zn	2	1	25	3000	3.0
Alginate gel [42]	Mg	0.5	0.5	25	180	0.09

**Table 3:** Unified benchmarking of recent biomimetic flow battery strategies.

System/Strategy	*J* mA cm^−2^	CE (%)	EE/VE (%)	Capacity Retention/Cycling
FMN//Fe(CN)_6_ AORFB(bio-mimetic flavin mononucleotide) [49]	10–80	>99	—	200 cycles @ 80 mA cm^−2^
Alkaline quinone RFB (Alkaline quinone flow battery) [51]	100	99	84	100 cycles; ≈0.1%/cycle decay
Solvation-regulated AORFB (DHAQ system) [50]	10	—	—	100 cycles @ 10 mA cm^−2^ (TMA^+^ 0–4.5 M gradient); decay rate from 5.34% day^−1^ → 0.65% day^−1^ (0.1 M DHAQ)
Amino acid-functionalized phenazine (nearly neutral AORFB) [52]	20	—	—	An extremely low-capacity fade rate of 0.5% per year
Polymer-based aqueous RFB (non-corrosive, low-cost) [48]	40	—	75–80	After 10,000 consecutive cycles at 20 mA cm^−2^ (no pumping), the remaining capacity is 80%
sPIM-SBF ion sieving membrane (AORFB) [30]	100	—	≈78–79	2100 cycles
CTS–Cu^2+^ chitosan composite membrane (alkaline Zn-RFB) [31]	200–320	97.41	≈80	200 cycles @ 200 mA·cm^−2^
Self-healing AEM (Diels–Alder network, pH-7 RFB) [32]	10	≈97	>79	100 cycles
PDA@SPEEK coating (VRFB) [33]	80	98.5	—	70% @ 150 cycles, 80 mA·cm^−2^

## Data Availability

No new data were created or analyzed in this study. Data sharing is not applicable to this article.
